# Assessment tools for patient-reported outcomes in multiple myeloma

**DOI:** 10.1007/s00520-023-07902-4

**Published:** 2023-06-30

**Authors:** Ting Wang, Qin Lu, LeiWen Tang

**Affiliations:** 1 Department of Haematology, Sir Run Run Shaw Hospital, Zhejiang University School of Medicine, Hangzhou City, Zhejiang Province China; 2 Department of Nursing, Sir Run Run Shaw Hospital, Zhejiang University School of Medicine, Hangzhou City, Zhejiang Province China

**Keywords:** Multiple myeloma, Patient-reported outcomes, Assessment tools, Nursing care

## Abstract

**Background:**

Patients with multiple myeloma experience severe symptom burden. Patient participation in self-reporting is essential as medical staff’s assessment of patient symptom severity is often lower than patient self-reporting. This article reviews patient-reported outcome (PRO) assessment tools and their application in the field of multiple myeloma.

**Results:**

The European Organization for Research and Treatment of Cancer Quality of Life Questionnaire Core 30 (EORTC QLQ-C30) is the universal patient-reported outcome assessment tool most frequently used to evaluate the life quality in people with multiple myeloma. Among the specific patient-reported outcome assessment tools, the European Organization for Research and Treatment of Cancer Quality of Life Questionnaire-Multiple Myeloma Module (EORTC QLQ-MY20), the Functional Assessment of Cancer Therapy-Multiple Myeloma (FACT-MM), and the M.D. Anderson Symptom Inventory-Multiple Myeloma Module (MDASI-MM) are the most widely used, with some scholars using the EORTC QLQ-MY20 as a calibration correlate for scale development.

Most current assessment instruments were developed using classical measurement theory methods; future researchers could combine classic theory tests and item response theory to create scientific assessment instruments.

In addition, researchers select the appropriate assessment tool based on the purpose of the study. They can translate high-quality assessment tools into different languages and consider applying them more often to assessing multiple myeloma patients. Finally, most existing PROs focus on measuring life quality and symptoms in people with multiple myeloma, with less research on outcomes such as adherence and satisfaction, thus failing to comprehensively evaluate the patient treatment and disease management.

**Conclusions:**

Research has shown that the field of PROs in multiple myeloma is in an exploratory phase. There is still a need to enrich the content of PROs and develop more high-quality PRO scales for multiple myeloma based on the strengths and weaknesses of existing tools. With the successful advancement of information technology, PROs for people with multiple myeloma could be integrated with electronic information systems, allowing patients to report their health status in real time and doctors to track their condition and adjust their treatment, thereby improving patient outcomes.

## Introduction

Multiple myeloma, a proliferative clonal malignant plasmocyte disease, is the second most common hematological malignancy [1, 2]. The number of new cases of multiple myeloma in the USA is 35,000 annually, and approximately 588,000 worldwide [3]. Multiple myeloma cases increased by 126% worldwide from 1990 to 2016, and deaths by 94% [4]. Studies have shown that people with multiple myeloma have experienced a more significant symptom burden in recent years than patients with other hematological malignancies [5]. With the development of autologous stem cell transplantation technology and the introduction of new drugs, the survival rate of multiple myeloma has improved. Patients have to cope with ongoing disease symptoms and repeated treatment toxicities. Therefore, it is important to focus on the quality of life as one of the priorities.

Currently, disease assessment relies on objective indicators such as physical examination, laboratory tests, and imaging, which may underestimate the impact of the disease on the individual and overestimate the effectiveness of medical interventions [6]. Patient-reported outcomes (PROs) are derived directly from the patient’s subjective assessment, including reports of their symptoms, health-related quality of life, daily and social functioning, and patient satisfaction, which do not require interpretation by medical staff or any other person [7]. In contrast, PROs provide a patient’s eye view of their condition. Providers can provide patients with timely, personalized medical care based on patient-reported outcomes. Patient-reported outcomes can provide evidence for medical decision-making and health policy development and as a reference indicator for symptom monitoring.

This article reviews the main components, psychometric characteristics, applications, and limitations of commonly used tools for assessing PROs in multiple myeloma. It discusses future directions for PROs in the clinical field, intending to generate helpful evidence.

## Multiple myeloma universal patient-report outcome assessment tool

### Medical Outcome Study Short Form 36

The Medical Outcome Study Short Form 36 (SF-36) [8] is a brief health questionnaire based on the Medical Outcome Study (MOS) in the USA. It is primarily used in clinical research, health policy evaluation, and general population surveys. The SF-36 is one of the most widely used standardized quality-of-life measurement instruments internationally and has been translated into more than 40 languages. The SF-36 can be collected by self-assessment, another assessor, or telephone questioning and takes about 15 min to complete. However, it has many entries and a significant response burden, so some scholars have developed a shortened form, consisting of 12 and 8 entries for the SF-12 and SF-8, respectively, to shorten the completion time.

### European Quality-of-Life Five-Dimension Scale

The European Quality-of-Life Five-Dimension Scale (EQ-5D) [9] is a multidimensional health-related quality-of-life scale developed by the European Research Group on Quality of Life in 1990, consisting of 2 components, the Health Description System and the Visual Analogue Scale (EQ-5D Visual Analogue Scale, EQ-VAS). The Health Descriptor System reflects three areas of physical health, social functioning, and mental health and can be completed in 10 min. The EQ-VAS is used to measure the overall health status of the subject. The EQ-5D Scale was used by Plesner et al. [10] to assess the quality of life in a clinical trial of a drug for multiple myeloma. The unique to this tool is that it can be used to estimate quality-adjusted life year (QALY) gains in health economic evaluation and has been used in several countries, including the UK, Germany, and the USA. However, it measures the subject’s condition on the same day, and its retest reliability is low when the retest interval is more than 1 week, so the scale is more suitable for measuring the quality of life in chronic diseases.

### Edmonton Symptom Assessment System

In 1991, Canadian researchers Bruera and colleagues developed the Edmonton Symptom Assessment System (ESAS), assessing the incidence and severity of physical and mental symptoms among cancer patients with advanced and palliative diseases [11]. It has nine required symptoms, such as discomfort, tiredness, nausea, and loss of appetite, plus one optional symptom. Chinese academics 2015 revised it and added “itchy skin” to the Chinese translation, which scored 0.72 on Cronbach’s alpha [12]. However, it was not tested for validity. Ebraheem and colleagues explored factors affecting symptoms in multiple myeloma patients receiving autologous stem cell transplants using the ESAS [13]. Despite the scale’s widespread use, Bruera does not specify a timeframe for the assessment, and most scholars use the “last 24 h” for assessment. ESAS is simple, easy to perform, and equally applicable to chronic non-cancer patients with palliative care needs [14].

Furthermore, the assessment results can be translated into symptom trends and accurately determine the severity of a patient’s symptoms. Its limitations are that individual entries are not accurate, and some scholars have revised “appetite” to “loss of appetite” and reordered and regrouped the nine symptoms [15]. The scale is not comprehensive enough as it only contains nine symptoms of cancer patients. Researchers should use this scale in conjunction with other scales to evaluate the full range of symptoms in cancer patients.

### Memorial Symptom Assessment Scale

The Memorial Sloan-Kettering Cancer Center in the USA created the Memorial Symptom Assessment Scale (MSAS), which consists of 32 entries, developed in 1994 [16] to assess the physical and psychological symptoms, and global distress index in cancer patients over the past week. Twenty-four entries assess symptoms’ incidence, severity, and distress, while eight entries assess the severity and distress of symptoms. Cheng et al. [17] showed that the internal consistency of the Chinese version of the MSAS was 0.79–0.87, and the content validity was 0.94 in assessing the symptoms of Chinese cancer patients. The scale is comprehensive and covers 32 common cancer symptoms, but it is time-consuming to complete, and the scoring rules are complex.

Based on this, some researchers revised the Memorial Symptom Assessment Scale Short Form (MSAS-SF) and the Condensed Memorial Symptom Assessment Scale (CMSAS), measuring the incidence and distress of 32 and 14 symptoms, respectively [18]. The Chinese versions of the MSAS-SF and CMSAS Cronbach’s alpha coefficients ranged from 0.84 to 0.91 and 0.79 to 0.87, respectively, with good construct validity [19]. Chen et al. used the Chinese version of the MSAS-SF to investigate symptom clusters in outpatients with multiple myeloma. They discovered three symptom clusters: the psychological symptom cluster, the painful dry mouth and sleep difficulty symptom cluster, and the fatigue symptom cluster. There was a link between the symptom clusters and the clinical symptoms [20]. No MSAS-specific module for multiple myeloma is currently available.

### Hematological Malignancy Specific Patient-Reported Outcomes Measure

In 2020, Goswami et al. developed the Hematological Malignancy Specific Patient-Reported Outcomes Measure (HM-PRO) [21] and applied it to all hematological malignancies. The scale contains two subscales, scale A and scale B. Scale A assesses the impact of the disease and associated treatments on the patient’s quality of life. Scale B evaluates the severity of symptoms and side effects of treatment over the previous 3 days.

The HM-PRO is the first universal quality of life and symptom measurement scale for hematological malignancies. It was developed using a combination of classic theory tests and item response theory, and it has excellent reliability and responsiveness. The HM-PRO not only permits early identification of the factors that have the most significant impact on patients during treatment and disease, but also allows for evaluating the effectiveness of treatments and monitoring dynamic changes in patients’ life quality [22]. Due to the late development of the scale, neither domestic nor international research has yet to use it. As a result, its scientific validity can be validated through large-scale studies in the future.

## Multiple myeloma-specific patient-reported outcome assessment tool

### European Organization for Research and Treatment of Cancer, the Quality-of-Life Questionnaire-Multiple Myeloma Module

In 1999, Stead et al. developed the European Organization for Research and Treatment of Cancer Quality-of-Life Questionnaire-Multiple Myeloma Module (EORTC QLQ-MY24) [23]. The twenty-four items span five dimensions: body image, social support, future perspective, side effects of treatment, and disease symptoms. In 2007, Cocks et al. [24] removed the social support dimension due to its ceiling effect and revised the remaining four dimensions into the EORTC QLQ-MY20, designed to assess the quality-of-life status of people with multiple myeloma over the past week.

Notably, the EORTC QLQ-MY20 is a multiple myeloma-specific scale developed based on The Quality of Life Questionnaire Core 30 (QLQ-C30) [25], which needs to be combined with the EORTC QLQ-C30 to measure the quality of life of people with multiple myeloma. Li et al. [26] applied the EORTC QLQ-MY20 scale to investigate the life quality of Chinese people with multiple myeloma. It showed that people with multiple myeloma have a low quality of life and are closely related to their physical condition and depression and anxiety, disease stage, and whether the diagnosis was made early. The most widely utilized quality-of-life scale in multiple myeloma is the EORTC QLQ-MY20, which is comprehensive, translated, and used in many nations [27–30]. However, when combined with the QLQ-C30, the number of entries is excessive and may lead to a patient response burden [31]. Future researchers can make the scale more streamlined and optimized to meet our needs.

### Functional Assessment of Cancer Therapy-Multiple Myeloma

Wagner et al. developed the Functional Assessment of Cancer Therapy-Multiple Myeloma (FACT-MM) in 2012 [32]. The scale consists of the FACT-General Scale (FACT-G) [33] and multiple myeloma-specific modules designed to assess patients’ health states and life quality during the previous week. The multiple myeloma-specific modules consist of 14 entries, including bone pain, physical pain, fatigue, difficulty walking, and weight gain.

Gupta et al. utilized the FACT-MM to investigate how treatment adherence impacts reported outcomes in patients with multiple myeloma [34]. Due to its comprehensiveness and focus on psychiatric symptoms, the FACT-MM is the most popular quality-of-life instrument for multiple myeloma patients after the EORTC QLQ-MY20. However, the measurement could have been more precise because of the study population’s racial diversity, young age, and the fact that 38% of patients were in remission. To investigate the applicability of the scale to patients with multiple myeloma of various races, ages, and disease stages, in the future, may increase the population.

### M.D. Anderson Symptom Inventory-Multiple Myeloma Module

The M.D. Anderson Symptom Inventory-Multiple Myeloma Module (MDASI-MM)[31] is a specific version of the M.D. Anderson Symptom Inventory (MDASI) [35], developed by Jones et al. at the Anderson Cancer Center in 2013 to assess the severity of symptoms and their interference with daily life in the past 24 h in people with multiple myeloma. Several authors [36] have investigated that the MDASI-MM can be a sensitive measure of symptom burden in relapsed or refractory multiple myeloma. Wang et al. [37] used the scale to explore the relationship between inflammatory markers and symptom burden during autologous stem cell transplantation in people with multiple myeloma and confirmed a correlation between the two.

Unlike the EORTC QLQ-MY20 and FACT-MM, which include symptom assessment but were developed based on a framework of health-related quality of life, the MDASI-MM was developed based on a framework of symptom assessment and is an accurate symptom assessment scale. The MDASI-MM has concise entries that capture the most critical symptom issues for patients with minimal response burden and is widely recognized. It is clinically applicable and supports an interactive verbal response system for symptom assessment [37]. It also allows repeated measurement of patient symptom severity [38], providing an additional advantage for longitudinal studies of symptoms. In addition, the MDASI specificity module was developed based on the MDASI core module and facilitated the comparison of symptom incidence and severity across cancers. The sample size could be increased for future multi-center research to address the drawback of the included samples from the same cancer center. Secondly, it could be cross-culturally adapted to allow for accurate symptom assessment of multiple myeloma patients from different countries and ethnicities.

### Myeloma Patient Outcome Scale

In 2015, Osborne et al. created the Myeloma Patient Outcome Scale (MyPOS), based on the Palliative Care Outcome Scale, to evaluate the quality of life of multiple myeloma patients in the clinical setting by engaging patients to recall health problems from the past 7 days [39]. Ramsenthaler et al. [40] applied the scale to measure the quality of life trajectories and palliative care issues in 238 people with multiple myeloma and showed that a decline in quality of life was associated with high levels of symptoms, pain, and anxiety and that clinical attention should be paid to patient-reported symptoms and psychosocial health to identify patients with palliative care needs.

Unlike the EORTC QLQ-MY20 and FACT-MM, which are primarily applicable to clinical trials, the MyPOS is the first multiple myeloma scale explicitly developed for clinical care, covering medical support items to assess patient understanding of the disease and satisfaction with treatment. MyPOS had a 91.6% response rate for the sexual functioning items in the original authors’ study. Despite this, low response rates to sexual functioning items may occur due to cultural differences in different countries. In addition, the healthcare support dimension’s low reliability and ceiling effect limit its widespread use, and continued refinement is needed to improve item response rates and reliability. Currently, the scale has only been translated into German [41].

### Multiple Myeloma Symptom and Impact Questionnaire

American researchers Gries et al. [42] created the Multiple Myeloma Symptom and Impact Questionnaire (MySIm-Q) in 2021 to assess the symptoms experienced by patients with active multiple myeloma treated with immunological drugs in the past week and their impact on daily life. For the first time, a leg pain entry was added to the pain dimension to assess pain symptoms associated with areas other than the back or legs in people with multiple myeloma. The MySIm-Q does not evaluate the adverse effects of the therapy and only concentrates on the symptoms linked to the disease itself. The MySIm-Q was developed using a combination of classic theory test and item response theory, incorporating ethnically diverse populations as study participants with good content validity. A limitation is that the scale has yet to be reported in other studies, as psychometric properties such as internal consistency and construct validity are being validated. The scale is promising given that it was developed specifically for people with multiple myeloma treated with immunological agents and that the treatment of multiple myeloma is currently in a new era of immunology where new immunotherapeutic approaches are receiving increasing scholarly attention.

## Discussion

The SF-36, EQ-5D, and EORTC QLQ-C30 are the universal patient-reported outcome assessment tools most frequently used to evaluate the life quality in people with multiple myeloma. In contrast, the ESAS and MSAS are commonly used to assess symptoms in people with multiple myeloma. The EORTC QLQ-MY20, FACT-MM, and MDASI-MM are the most widely used among the specific patient-reported outcome assessment tools. Some scholars use the EORTC QLQ-MY20 as a calibration correlate for scale development.

Table 1 shows the characteristics and psychometric properties of eight patient-reported outcome assessment tools for multiple myeloma.

**Table 1:** Assessment tools For patient-reported outcomes with multiple myeloma: characteristics and psychometric properties

Instrument and country of origin	Target group	Items and domains	Response options	Validity		Reliability		Responsiveness	Feasibility
				Content validity	Construct validity	Internal consistency	Reproducibility		
SF-36 [8]; the USA	Anyone	36 items; 8 domains: •Physical functioning (10) •Social functioning (2) •Role limitations due to physical health problems (4) • Bodily pain (2) •Mental health (5) •Role limitations due to emotional problems (3) •Vitality (4) •General health perceptions (6) •Reported health transition	Rated on 2 to 6 ordered categories	•Literature •Expert review •Review by patients •Pilot work	Demonstrated convergent and divergent validity through comparison with: • Nottingham health profile	Domains: ranged from *α* = 0.73 to 0.96	Time:2 weeks (Spearman correlation coefficients 0.60~0.80)	-	Time:15 minutes Reading level: unknown Made for clinical research, health policy evaluation, general population
EQ-5D [9]; European Quality-of-Life Group	Anyone	Part 1: EQ-5D-5L 5 items; 5 domains: •Mobility (1) •Self-care (1) •Usual activities (1) •Pain/discomfort (1) •Anxiety/depression (1) Part 2: EQ-VAS 1 item	Part 1: 5-point Likert scale (no problem/extreme problem) Part 2: 0~100-min scale (worse of health you can imagine/best of health you can imagine)	-	Demonstrated convergent and divergent validity through comparison with: •SF-36	Domains: ranged from *α* = 0.74 to 0.91	Time: 7 days (Spearman correlation coefficients 0.82)	-	Time: 10 minutes Reading level:unkonwn Made for any place
ESAS [11]; Canada	Patients with advanced and palliative cancer	10 items; 3 domains: •Physical (6) •Psychosocial (3) •Optional symptom (1)	10-point VAS (no/worst possible)	-	Demonstrated convergent validity through comparison with: •Functional Assessment of Cancer Therapy (FACT) • Memorial Symptom Assessment Scale (MSAS) • Brief Pain Inventory (BPI)	Overall: *α* = 0.79	Time:2 days (Spearman correlation coefficients 0.86) Time: 7 days (Spearman correlation coefficients 0.45)	-	Time:5 minutes Reading level: older patients have difficulty understanding Made for the clinical setting
MSAS [16]; the USA	Cancer patients	32 items; 2 domains: •Physical (28) •Psychosocial (4)	The incidence and severity of symptoms: 4-point Likert scale (rarely or slight/almost constantly or very severe) Global Distress Index: 5-point Likert scale (not at all/very much)	-	-	Domains: ranged from *α* = 0.58 to 0.88	-	-	Time: unknown Reading level: unknown Made for the clinical setting
HM-PRO [21]; the UK	Patients with hematological malignancies	Scale A: 24 items; 4 domains: •Physical (7) •Social (3) •Emotional (11) •Eating and drinking habits (3) Scale B: 18 items; 1 domain: •Disease symptoms and side effects of treatment (18)	Scale A: 3- point Likert scale (not at all/a lot) Scale B: 3-point Likert scale (not at all/severe)	•Literature •Cognitive debriefing interviews •Expert review	Demonstrated convergent validity through comparison with: •EORTC QLQ-C30 • FACT-G	Scale A: *α* = 0.95 Scale B: *α* = 0.85	Between 1 and 7 days apart : Scale A: intraclass correlation coefficients = 0.93 Scale B: intraclass correlation coefficients = 0.91 Interrater reliability: Scale A: correlations ranged from *r* = 0.58 to 0.86 Scale B: correlations ranged from *r* = 0.49 to 0.72	-	Time: 5 minutes to complete paper version, 6.5 min to complete electronic version Reading level:unkonwn Acceptability: showed preference toward completing the electronic version Made for the clinical setting
EORTC QLQ-MY20 [24]; the UK	Multiple myeloma	EORTC QLQ-C30: 30 items; 15 domains: •Physical (5) •Role (2) •Cognitive (2) •Emotional (4) •Social (2) •Fatigue (3) •Pain (2) •Nausea and vomiting (2) •General health status (2) •Single-item symptom (6) MM-specific modules: 20 items; 4 domains: •Body image (1) •Future perspective (3) •Side effects of treatment (10) •Disease symptoms (6)	4-point Likert scale (none/extremely)	•Literature •Expert review •Review by patients •Pilot work	Demonstrated convergent and divergent validity through comparison with: •EORTC QLQ-C30	Domains: ranged from *α* = 0.76 to 0.89	Time: 3 months, 6 months (internal consistency ranged from *α* = 0.70 to 0.92)	Changes in quality of life over time are expected as a patient’s condition changes	Time: 12 minutes Reading level:unkonwn Made for the clinical setting
FACT-MM [32]; the USA	Multiple myeloma	FACT-G: 27 items; 4 domains: •Physical (7) •Functional (7) •Social and family (7) •Emotional (6) MM-specific modules: 14 items	5-point Likert scale (not at all/very much)	•Literature •Expert review •Review by patients •Reviewed by the FACT Multilingual Translation Team	-	Domains: ranged from *α* = 0.79 to 0.89	-	-	Time: unknown Reading level: unknown Made for the clinical setting
MDASI-MM [31]; the USA	Multiple myeloma	MDASI: 19 items; 2 domains: •Core symptom (13) •Symptom interference (6) MM-specific modules: 7 items	10-point scale (not present or did not interfere/as bad as you can imagine or interfered completely)	•Literature •Cognitive debriefing interviews	Demonstrated convergent and divergent validity through comparison with: •EORTC QLQ-C30 •EORTC QLQ-MY20 •Eastern Cooperative Oncology Group(ECOG)	Domains: ranged from *α* = 0.85 to 0.91	-	MDASI-MM subscale change scores were significant for patients with worsening ECOG PS, but were not significant for patients with stable/improving ECOG PS. Demonstrate the ability to capture significant changes in patient symptoms over time before and 7 days after hematopoietic stem cell transplantation	Time: unknown Reading level: unknown Made for: the clinical setting
MyPOS[39]; the UK	Multiple myeloma	30 items; 3 domains: •Symptom functioning (12) •Emotional response (11) •Healthcare support (7)	5-point Likert scale (never or none/always or severe)	•Literature •Cognitive debriefing interviews •Expert review	Demonstrated convergent and divergent validity through comparison with: •EORTC QLQ-C30 •EORTC QLQ-MY20 •Eastern Cooperative Oncology Group(ECOG) •Factor analysis	Domains: ranged from *α* = 0.64 to 0.84	-	-	Time: 7 minutes 19 seconds Reading level:85% completed Secondary school or greater education Acceptability:73.4% without assistance Made for the clinical setting
MySIm-Q [42]; the USA	Treatment of active multiple myeloma with immunological drugs	17 items; 8 domains: •Pain (3) •Neuropathy (1) •Fatigue (4) •Digestive (1) •Cognitive (2) •Activity (3) •Social (1) •Emotional (2)	5-point Likert scale (never or none/always or severe)	•Literature •Hybrid concept elicitation •Cognitive debriefing interviews	-	-	-	-	Time: unknown Reading level: unknown Made for clinical trials

*SF-36* Medical Outcome Study Short Form 36; *EQ-5D* European Quality-of-Life Five-Dimension Scale; *ESAS* Edmonton Symptom Assessment System; *MSAS* Memorial Symptom Assessment Scale; *HM-PRO* Hematological Malignancy Specific Patient-Reported Outcome Measure;

*EORTC QLQ-MY20* European Organization for Research and Treatment of Cancer Quality of Life Questionnaire-Multiple Myeloma Module; *FACT-MM* Functional Assessment of Cancer Therapy-Multiple Myeloma;

*MDASI-MM* M.D. Anderson Symptom Inventory-Multiple Myeloma Module; *MyPOS* Myeloma Patient Outcome Scale; *MySIm-Q* Multiple Myeloma Symptom and Impact Questionnaire

### Applications

Most patients with MM require multiple treatment options and have cumulative toxicity and a high symptom burden. In contrast, symptom management is integral to care management, and effective symptom management is essential for improving a patient’s quality of life. Ramsenthaler et al. [43] used MyPOS and EORTC QLQ-C30 and QLQ-MY20 to investigate symptoms and quality of life in patients with multiple myeloma and showed that an average of 7.2 symptoms were present per patient to painful multiple myeloma patients with high symptom burden and low HRQOL in late and early disease. Therefore, the focus in the clinic should be on assessing patient-reported outcomes, enhancing patient symptom management, and improving quality of life. In summary, PROs can investigate patients’ existing symptom problems, monitor changes in symptoms, and help healthcare professionals develop comprehensive interventions to improve the quality of care.

PROs can help healthcare professionals gather health information, such as daily symptoms and drug toxicity reactions, and develop more standardized and personalized treatment protocols through a comprehensive assessment of treatment effectiveness. The introduction of PRO evaluation in clinical trials and practice, and as an endpoint indicator in clinical trials, is advocated in the PROs in Haematology guidelines.

In addition, Dubois scholars found PRO data to be of prognostic value for patient survival when bortezomib was administered to 202 patients with multiple myeloma [44].

### Limitations

Most current assessment tools have been developed using classical measurement theory methods, with limitations such as identical test items, dependence on sample size, broad and single-error indicators, and imprecise reliability estimates [45]. Secondly, there are still limitations to using multiple myeloma-specific scales worldwide due to language limitations, with most studies using universal scales. Thirdly, most of these PRO assessment tools were developed before developing newer therapies (e.g., monoclonal antibodies, CAR-T therapies, T cell engagers) with different side effect profiles. Furthermore, patients may have various quality-of-life needs during the maintenance phase of treatment after autologous transplantation, and these tools cannot cover all of them. Finally, most of the available PROs focus on measuring the quality of life and symptoms in patients with multiple myeloma, with less research on outcomes such as adherence and satisfaction, thus failing to assess patient treatment and disease management comprehensively.

### Future perspectives

It is recommended that future researchers combine classical test theory and item response theory to develop multiple myeloma-specific scales that cover various dimensions such as physical, psychological, social, satisfaction, and treatment adherence, thereby helping healthcare professionals to assess patients and take targeted measures and care comprehensively. In addition, researchers should select appropriate assessment tools according to the purpose of the study. Existing high-quality assessment tools can be translated into different languages and considered for greater use in assessing patients with multiple myeloma. Finally, researchers can combine advances in information technology and artificial intelligence to develop management systems suitable for patients with multiple myeloma, combining electronic versions of assessment tools with patient management systems to enable the sharing of doctor-patient information and provide a realistic basis for patient participation in treatment decisions and facilitate patient management.

## Conclusion

Research has shown that the field of PROs in multiple myeloma is in an exploratory phase. There is still a need to enrich the content of PROs and develop more high-quality PRO scales for multiple myeloma based on the strengths and weaknesses of existing tools. With the successful advancement of information technology, PROs for people with multiple myeloma could be integrated with electronic information systems, allowing patients to report their health status in real time and doctors to track their condition and adjust their treatment, thereby improving patient outcomes.
